# Infectious complications of probiotic use: A matched case–control study

**DOI:** 10.1017/ice.2021.261

**Published:** 2022-10

**Authors:** Florissa S. Tom, Kendall J. Tucker, Caitlin M. McCracken, Jessina C. McGregor, Sara J. Gore

**Affiliations:** 1 Oregon Health & Science University-Portland State University School of Public Health, Portland, Oregon; 2 Oregon State University College of Pharmacy, Corvallis, Oregon; 3 Oregon Health & Science University, Portland, Oregon

## Abstract

In this matched case–control study, we sought to determined the association between probiotic use and invasive infections caused by typical probiotic organisms. The odds of probiotic use in cases were 127 times the odds of probiotic use in controls (95% CI, 6.21–2600). Further research into these rare but severe complications is needed.

Probiotics may be beneficial for specific indications; meta-analyses have demonstrated that probiotics can reduce the risk of *Clostridioides difficile* infection in adults by 60% and can reduce the risk of antibiotic-associated diarrhea in children by 55%.^1,2^ Inpatient probiotic treatment is prevalent and increasing, with recent studies demonstrating that 0.3%–8.5% of inpatients receive probiotics in the United States.^3,4^ Despite the increased use of probiotics, safety concerns persist, especially in patients who are immunocompromised or have impaired gastrointestinal tract integrity.^5,6^ Evidence supporting probiotic safety primarily involves ecologic data, which demonstrate that rates of infection with probiotic organisms remain stable as probiotic use increases.^7^ These ecologic analyses are hindered by lack of individual-level data, estimates of probiotic exposure, and low sample sizes due to low infection rates. Systematic reviews and meta-analyses from clinical trials are limited by sporadic adverse event reporting.^8^ Given the low incidence of infections with probiotic organisms (<1% of all positive blood cultures^7^), most accounts of infections are case reports and series.^9^ In a recent case–control study performed in a pediatric intensive care unit, researchers identified whole-genome–based phylogenetic and epidemiologic linkages between probiotics and *Lactobacillus* bloodstream isolates in 6 patients, showing a significantly higher risk of *Lactobacillus* bacteremia in the patients who received probiotics.^10^ However, no studies have assessed the odds of developing infectious complications of probiotics in the adult inpatient population. We evaluated the association between probiotic treatment and invasive infections caused by probiotic organisms among hospitalized adults.

## Methods

We conducted a matched case-control study of adults aged ≥18 years admitted to Oregon Health & Science University (OHSU), an academic hospital located in Portland, Oregon, between October 1, 2015, and August 20, 2019. Study participants were identified using the Pharmacy Research Repository (PHARR), a repository from the OHSU electronic health record (EHR) system.

Cases were defined as patients with positive cultures from normally sterile sites (ie, blood, cerebral spinal fluid, ascitic fluid, or pleural fluid) with common probiotic organisms (eg, *Lactobacillus, Lactococcus, Bifidobacterium,* or *Saccharomyces*) found in probiotic capsules and known to occur in commercial kefir products. We included patients with multiple sets of blood cultures, a positive culture from sterile fluid or tissue with signs or symptoms of infection, or 1 set of positive blood cultures with signs or symptoms of infection and a predisposing condition (impaired gastrointestinal tract integrity or immunosuppression). We reviewed medical records to verify case status, which was assigned independently by 2 coinvestigators. Discrepancies were resolved by the principal investigator, and cases were excluded if they did not meet the culture requirements. Also, 3 controls were randomly selected and matched to cases by admission date (within 7 days of the case’s date of admission), length of stay (at least the duration of the case’s time at risk), and primary inpatient team specialty.

The EHRs were reviewed to capture demographics, comorbidities, probiotic exposure, and potential confounders, including solid-organ and bone-marrow transplant, malignancy, immunosuppression level, and abdominal surgical history during each patient’s time at risk. Time at risk for cases was defined as duration from admission date to the index culture collection date. Time at risk for controls was at least the same duration as their matched cases, starting from the date of admission. All data extracted were collected in the OHSU instance of Research Electronic Data Capture (REDCap).

The primary exposure of interest was use of probiotics. Probiotic use was defined as administration of *Lactobacillus* capsules, probiotic yogurt, or probiotic kefir documented in the EHR. Data were also collected for patient demographics and suspected confounding variables. Immunosuppression was defined as severe (bone-marrow or solid-organ transplant patients or patients with liquid or solid tumors) or mild to none (all other patients). Gastrointestinal (GI) integrity was determined using *International Classification of Disease, Tenth Revision* (ICD-10) codes for the following conditions: GI artificial opening, perforation, fistula, ulcer, mucositis, complication of GI device, diagnosis of Crohn’s disease, ulcerative colitis, or other noninfective colitis. Probiotic use, abdominal surgery, endoscopy, central venous catheter use, and antimicrobial use were only evaluated within the patient’s time at risk. A descriptive statistical analysis was performed to characterize the study population. We used a mixed effects logistic regression model to measure the odds ratios (ORs) and 95% confidence intervals (CIs) for the association between probiotic use and subsequent infection. This analytic approach allowed for appropriate handling of the matched study design and the sparse distribution of exposure across strata. Statistical analysis was performed using STATA version 15 software (StataCorp, College Station, TX).

## Results

Of the 31 patients who met the case criteria for our study, we identified matched controls for all but 3 (excluded because adequate controls could not be identified), for a total of 112 study participants (28 cases and 84 controls). The study population primarily consisted of white males with an overall average age of 56 years (Table 1). Moreover, 12 patients (11%) were prescribed probiotics with a median duration of 6.5 days (range, 5–9.5). The most common organism was *Lactobacillus* (68%) and the least common was *Saccharomyces* (4%). All 7 cases receiving *Lactobacillus* containing kefir or probiotic yogurt grew *Lactobacillus*. Using the Pearson’s χ^2^ test, we observed that cases were more likely to have had abdominal surgery within the past month or year (*P* = .008 and *P* = .013, respectively). In addition, the cases had significantly higher in-hospital mortality (*P* = .015) (Table 2). Controls were more likely to have chronic kidney disease (*P* = .005), to have undergone solid-organ transplantation (*P* = .010), and to be severely immunosuppressed (*P* = .022). Of 28 matched groups (consisting of 1 case and 3 controls), 21 groups were all unexposed to probiotics, 1 group included cases and controls all exposed to probiotics, and 6 groups had a combination of exposed and unexposed. Based upon the regression analysis, we estimated that the odds of probiotic use among those with invasive infections were 127 times greater than the odds of probiotic use among patients without invasive infections (95% CI, 6.21–2,600).

**Table 1.** Study Population^a^ Characteristics and Outcomes

Characteristic	Cases (n=28)	Controls (n=84)
Age, mean y (SD)	56 (18)	55 (15)
**Sex, no. (%)**		
Male	18 (64)	48 (57)
Female	10 (36)	36 (43)
**Race, no. (%)**		
American Indian/Alaskan	0 (0)	1 (1)
Asian	0 (0)	3 (4)
Black	1 (4)	3 (4)
White	26 (93)	72 (86)
More than one race	1 (4)	1 (1)
Unknown/unreported	0 (0)	4 (5)
**Ethnicity, no. (%)**		
Hispanic or Latino	0 (0)	8 (10)
Not Hispanic or Latino	28 (100)	75 (89)
Unknown	0 (0)	1 (1)
Diabetic, no. (%)	10 (36)	26 (31)
Chronic kidney disease, no. (%)	2 (7)	29 (35)
Cirrhosis, no. (%)	4 (14)	8 (10)
Inflammatory bowel disease, no. (%)	1 (4)	1 (1)
Human immunodeficiency virus, no. (%)	1 (4)	0 (0)
Impaired gastrointestinal mucosa	5 (18)	12(14)
Solid organ transplant	1 (4)	28(33)
Bone marrow transplant	3(11)	13(15)
Severe immunosuppression	8(29)	45(53)
Primary inpatient team, acute general care	14 (50)	42 (50)
Pathogen identified, No. (%)		
*Bifidobacterium* spp	6 (21)	
*Lactobacillus* spp	19 (68)	
*Lactococcus* spp	2 (7)	
*Saccharomyces* spp	1 (4)	
Duration of probiotic use, median (IQR), days	6(4-8)	6 (5-10)
Probiotic use, no. (%)	7 (25)	5 (6)
Culturelle	0	2
Kefir	3	2
Probiotic yogurt	4	1
Mortality during admission, no. (%)	4 (14)	2 (2)
Readmitted within 30 d after discharge, no. (%)	6 (21)	14 (17)

a The 3 excluded patients were white, aged 50, 57, and 61 years. All 3 patients grew *Lactobacillus* and 3 had received probiotics.

**Table 2.** Distribution of Risk Factors for Invasive Infections

Risk Factors	Cases (n=28)		Controls (n=84)	
	Prescribed Probiotics (n=7)	Not Prescribed Probiotics (n=21)	Prescribed Probiotics (n=5)	Not Prescribed Probiotics (n=79)
Length of stay, median d (IQR)	20 (8–29)	11 (6–23)	18 (16–20)	6 (4–18)
Time until bacteremia/time at risk, median d (IQR)	6 (1–10)	2 (1–7)	15 (15–15)	3 (1–10)
Duration of probiotic use, median d (SD)	6(4–8)	…	7 (6–11)	…
Impaired gastrointestinal mucosal integrity	3(43)	2(10)	1(20)	11(14)
Solid organ transplant, no. (%)	0 (0)	1 (5)	0 (0)	22 (28)
Bone marrow transplant, no. (%)	0 (0)	3 (14)	0 (0)	13 (17)
**Immunosuppression level, no. (%)**				
Severe immunosuppression	0 (0)	8 (38)	2 (40)	43 (54)
Mild/no immunosuppression	7 (100)	13 (62)	3 (60)	36 (46)
Abdominal surgery within 1 month, no. (%)	5 (71)	4 (19)	1 (20)	8 (10)
Endoscopy within 12 mo, no. (%)	3 (43)	3 (14)	0 (0)	13 (17)
Central venous catheter, no. (%)	5 (71)	10 (48)	4 (80)	43 (54)
Received antimicrobials, no. (%)	7 (100)	16 (76)	5 (100)	62 (79)

## Discussion

Our study identified a significant association between probiotic use and invasive infection with common probiotic organisms, with notable differences between cases and controls. Cases were significantly more likely to have had abdominal surgery and to die in the hospital. The association with abdominal surgery may be attributed to impaired gastrointestinal integrity or the use of postoperative probiotics. Important differences in the control group were significantly higher rates of organ transplantation and severe immunosuppression.

These unexpected findings may have stemmed from the matching criteria, which selected for younger patients with longer hospitalizations. Probiotic use in the controls may have been lower because our institution advises against probiotics use in immunocompromised populations. Notable limitations include the small sample size and sparse distribution of probiotic use, which prevented our analytic approach from accounting for suspected confounders beyond those used as matching variables. We were also unable to assess home probiotic use, which may have led to misclassification of exposure status. The patient population at our tertiary-care center is sick and complex, highlighted by the long lengths of stay in both arms, which limits the generalizability to hospitals with less complex populations.

In conclusion, this study observed significantly increased odds of probiotic use in patients with invasive infections with organisms commonly included in probiotics including *Lactobacillus, Lactococcus, Bifidobacterium,* and *Saccharomyces*. Overall, the risk of invasive infections was low compared to overall probiotic use because we identified only 31 invasive infections across the study period despite probiotic use in 6% of all admissions. Probiotic use is likely safe in the general population; however, certain patients may be at risk of complications. This finding adds to the existing literature on infectious complications of probiotics and lays the groundwork for larger studies investigating this risk.
